# Evaluation of Tryptophan and Its Metabolites in Predicting Disease Activation in Inflammatory Bowel Disease

**DOI:** 10.3390/jcm14031016

**Published:** 2025-02-05

**Authors:** Ali Karataş, Tarkan Karakan, Nergiz Ekmen, Yasemin Ünsal, Gülsüm Feyza Türkeş, Özlem Gülbahar, Mehmet Cindoruk, Mustafa Ergin, Güner Kılıç, Mehmet İbiş, Mehmet Arhan, İbrahim Doğan, Hasan Dağlı

**Affiliations:** 1 Department of Gastroenterology, Faculty of Medicine, Gazi University, Ankara 06560, Turkey; tkarakan@gazi.edu.tr (T.K.); dr_nergisekmen@hotmail.com (N.E.); mcindoruk@gazi.edu.tr (M.C.); mstfergn@hotmail.com (M.E.); gunerrkilic@gmail.com (G.K.); ibismehmet@yahoo.com (M.İ.); mehmetarhan@gmail.com (M.A.); idogan@gmail.com (İ.D.); 2 Department of Internal Medicine, Faculty of Medicine, Gazi University, Ankara 06560, Turkey; yaseminunsal93@gmail.com; 3 Department of Medical Biochemistry, Graduate School Of Health Sciences, Gazi University, Ankara 06560, Turkey; gfeyzaa90@gmail.com; 4 Department of Clinical Biochemistry, Faculty of Medicine, Ankara University, Ankara 06230, Turkey; 5 Department of Clinical Biochemistry, Faculty of Medicine, Gazi University, Ankara 06560, Turkey; ozengin@gazi.edu.tr (Ö.G.); hasandagli63@gmail.com (H.D.)

**Keywords:** inflammatory bowel disease, tryptophan, calprotectin

## Abstract

**Background and Aim**: Inflammatory bowel disease (IBD), which comprises ulcerative colitis (UC) and Crohn’s disease (CD), is characterized by chronic inflammation and fluctuating disease activity. This study aimed to evaluate serum tryptophan (TRP) and its metabolites as potential biomarkers for predicting disease activation in comparison to fecal calprotectin (FC). **Methods**: This prospective study included 115 patients (77 with UC and 38 with CD). Disease activity was assessed based on clinical and endoscopic findings. Serum TRP levels and their metabolites were measured using liquid chromatography–tandem mass spectrometry (LC-MS/MS), whereas FC levels were analyzed using an enzyme-linked immunosorbent assay (ELISA). **Results**: Serum TRP levels ≤ 11,328.41 ng/mL predicted disease activation with 72.1% sensitivity and 62.7% specificity, whereas FC levels ≥ 89.60 µg/g showed 84.2% sensitivity and 67.6% specificity. The TRP-to-C-reactive protein (CRP) ratio (TRP/CRP) demonstrated superior diagnostic accuracy, with an area under the curve (AUC) of 0.847. **Conclusions**: The TRP/CRP ratio is a novel and comprehensive approach for predicting disease activation in IBD patients. Although FC remains the gold standard, TRP and its metabolites provide valuable complementary insights. Further research is required to validate these findings in larger cohorts.

## 1. Introduction

Inflammatory bowel disease (IBD), encompassing ulcerative colitis (UC) and Crohn’s disease (CD), is a chronic condition characterized by unpredictable episodes of remission and activation. Its increasing prevalence worldwide presents a significant burden, affecting the quality of life of patients and posing a challenge to healthcare systems. Accurate assessment of disease activity is critical for guiding treatment strategies. However, traditional biomarkers, such as C-reactive protein (CRP) and sedimentation rate, often fail to correlate reliably with clinical symptoms or endoscopic findings, leaving a gap in the effective management of IBD.

Emerging research has highlighted the central role of tryptophan metabolism in IBD pathogenesis. Tryptophan, an essential amino acid, is metabolized predominantly via the kynurenine pathway and plays a pivotal role in regulating immune responses and maintaining gut homeostasis. The dysregulation of this pathway has been linked to chronic intestinal inflammation, impaired gut barrier function, and altered microbial diversity [1,2,3,4,5,6,7]. Kynurenine pathway metabolites, such as kynurenic acid and quinolinic acid, modulate key immune and inflammatory pathways by interacting with receptors, such as the aryl hydrocarbon receptor (AHR), influencing cytokine production and intestinal epithelial repair processes. This underscores the potential of tryptophan and its metabolites as biomarkers and therapeutic targets for IBD [8,9,10,11,12].

In addition to its immune-regulatory functions, tryptophan metabolism is intricately linked to gut microbiota. Gut microbes convert tryptophan to indole derivatives that are critical for intestinal homeostasis. These metabolites enhance mucosal integrity by stimulating interleukin-22 (IL-22) production, promoting tissue repair, and reducing inflammation. Moreover, downstream metabolites of tryptophan, such as serotonin, further contribute to gut physiology by influencing motility and secretion. The complex interplay between tryptophan metabolism, immune regulation, and gut microbial activity makes it a crucial area of study for understanding IBD pathogenesis and developing targeted interventions [12,13]. Singh et al. [14] in The American Gastroenterological Association (AGA) recommends the use of fecal calprotectin as a non-invasive biomarker in the management of ulcerative colitis. However, considerations, such as the requirement for stool sample collection, the potential for false positive and false negative results, variability in test performance, and limitations in detecting mild inflammation, should be taken into account.

In our study, the levels of tryptophan and kynurenine pathway metabolites, such as tryptophan, kynurenine, kynurenic acid, quinaldic acid, and 3-hydrokiskynurenin, were systematically determined in individuals with inflammatory bowel disease. Patients will be actively and inactively examined in the two groups, and we investigated whether there is a correlation between the levels of tryptophan and its metabolites and the clinical, laboratory, and endoscopic findings of disease activation and fecal calprotectin. Thus, the aim of this study was to determine disease activation earlier and more accurately and to evaluate the relationship between tryptophan metabolism and immune-regulatory pathways to identify novel therapeutic targets to modulate inflammation and restore gut homeostasis.

## 2. Materials and Methods

### 2.1. Study Design

This prospective, single-center, cross-sectional observational study was conducted to assess specific clinical, biochemical, and disease-related parameters in patients diagnosed with IBD. This study was conducted at the gastroenterology outpatient clinic of a tertiary care hospital between January 2020 and September 2021. Patients who met the inclusion criteria were invited to participate in this study. The participants were categorized into two groups based on their clinical evaluation at the time of enrollment: patients with active disease and those in remission. Informed consent was obtained from all patients prior to their inclusion in the study.

### 2.2. Patient Selection

The diagnosis of IBD, including UC and CD, was confirmed on the basis of a combination of clinical presentation, endoscopic findings, and histopathological evaluation. Patients aged 18–65 years who had been diagnosed with UC or CD were considered eligible for inclusion in the study.

### 2.3. Inclusion Criteria

- Confirmed diagnosis of UC or CD based on clinical, endoscopic, and histopathological findings.
- Age between 18 and 65 years.
- Informed consent provided.

### 2.4. Exclusion Criteria

- Concurrent infections or other inflammatory conditions, including rheumatoid arthritis, systemic lupus erythematosus, ankylosing spondylitis, and psoriatic arthritis.
- The use of medications known to affect tryptophan levels, such as corticosteroids, antidepressants (e.g., selective serotonin reuptake inhibitors), or immunomodulators, within the last month.
- The presence of comorbid medical conditions, such as diabetes mellitus, hypertension, or coronary artery disease, that may influence inflammatory or metabolic profiles.
- Recent antibiotic use (within the last 15 days) that can alter gut microbiota and tryptophan metabolism.
- Active autoimmune diseases (e.g., multiple sclerosis) diagnosed as per ACR criteria.
- Dietary habits potentially affecting tryptophan levels (e.g., high protein intake or restrictive diets) were assessed but not controlled. Patient diets were not included in this study to ensure that the findings reflect real-world metabolic variations and dietary influences on tryptophan metabolism. This approach aimed to preserve the ecological validity of the study and allow for the generalizability of the results to broader patient populations.

### 2.5. Evaluation of Disease Activity

Disease activity was assessed using validated scoring systems:

- The Partial Mayo Score was used for UC. Partial Mayo Score (≥2 for active disease).
- The Harvey–Bradshaw Index (HBI) was used for CD. Harvey–Bradshaw Index (≥5 for active disease).
- These scores allowed for the stratification of patients into active disease and remission groups.

### 2.6. Measurement of Fecal Calprotectin

Fecal calprotectin, a marker of intestinal inflammation, was measured using an enzyme-linked immunosorbent assay (ELISA). A fully automated Alegria stool calprotectin analyzer (Orgentec Diagnostics, GmbH) was used for these measurements. Stool samples were collected using specialized sample collectors provided to patients during their clinic visits. Following collection, the samples were stored at −80 °C until analysis.

### 2.7. Sample Preparation

Fecal calprotectin was extracted using standardized fecal extraction tubes. After thorough preparation, the extracted samples were analyzed. The results were automatically generated and recorded for subsequent statistical analyses.

### 2.8. Measurement of Serum Tryptophan and Its Metabolites

The kynurenine (KYN) pathway was analyzed to measure tryptophan (TRP) and its downstream metabolites, including:

- Kynurenine (KYN).
- Kynurenic Acid (KYNA).
- Picolinic Acid (PA).
- Quinolinic Acid (QA).
- 3-Hydroxykynurenine (3OHKYN).

#### 2.8.1. Sample Preparation and LC-MS/MS Analysis

Analysis of KYN metabolites was performed with an LCMS/MS device (Dionex UltiMate™ 3000 UHPLC and TSQ Quantum Access MAX Triple Stage Quadrupole Mass) as a result of the modifying of Tömösi et al.’s method [15].

▪ Serum (100 μL) was mixed with 370 μL acetone–methanol containing 10 μL internal standards (TRP, KYNA, PA, QA, and 3OHKYN).
▪ Vortexed for 60 s and incubated at −20 °C for 15 min.
▪ Centrifuged at 12,000× *g* for 15 min at 4 °C.
▪ The supernatant (400 μL) was transferred to tubes, split, and evaporated under nitrogen atmosphere.
▪ The mixture was derivatized with *n*-butanol–acetyl chloride (9:1, *v*/*v*) and incubated at 60 °C for 1 h.
▪ Samples were reconstituted in 100 μL eluent for LC-MS/MS analysis.

#### 2.8.2. LC-MS/MS Settings and Method Validation

▪ Chromatography was performed using an Agilent 1260 Infinity II system coupled with an Thermo TSQ Quantum Access MAX Triple Stage Quadrupole Mass Spectrometer (Waltham, MA, USA).
▪ The analytes were separated using a reverse-phase C18 column (Thermo Hypersil Gold 2.1 × 10mm, 3 µm, Waltham, MA, USA).
▪ The mobile phases consisted of water with 0.1% formic acid (A) and acetonitrile with 0.1% formic acid (B).
▪ A gradient elution program was applied: 0–2 min (5% B), 2–8 min (30% B), 8–12 min (50% B), 12–14 min (5% B) at a flow rate of 0.3 mL/min.
▪ Ionization was conducted in the positive electrospray ionization (ESI+) mode with a capillary voltage of 3.5 kV and a gas temperature of 300 °C.

Multiple reaction monitoring (MRM) mode was used for quantification with transitions:

▪ TRP: *m*/*z* 205.1 → 188.1
▪ KYN: *m*/*z* 209.1 → 146.0
▪ KYNA: *m*/*z* 190.1 → 144.0
▪ QA: *m*/*z* 168.1 → 122.0
▪ PA: *m*/*z* 124.0 → 78.0
▪ 3OHKYN: *m*/*z* 225.1 → 208.0

#### 2.8.3. Calibration and Validation

▪ Calibration curves were prepared by spiking the control serum with known analyte concentrations (range: 1–1000 ng/mL).
▪ Linearity, accuracy, precision, and recovery were validated as per FDA guidelines.
▪ The limits of detection (LOD) and quantification (LOQ) were determined for each analyte. (The detailed methodology is described in the Supplementary Materials).

### 2.9. Measurement of Serum Biochemical Parameters

Routine biochemical parameters, including:

▪ Erythrocyte sedimentation rate (ESR) (Sistat ESR, Ankara, Turkey).
▪ Total protein (Beckman Coulter AU5800, Inc., Brea, CA, USA).
▪ Albumin (Beckman Coulter AU5800, Inc., Brea, CA, USA).
▪ C-reactive protein (CRP) (Beckman Coulter AU5800, Inc., Brea, CA, USA).
▪ Ferritin (Beckman Coulter DXI800, Inc., Brea, CA, USA) was measured at the Central Biochemistry Laboratory of the hospital. State-of-the-art analyzers were used for these evaluations. The blood samples were processed immediately after collection to ensure accuracy. Data were recorded at the time of sampling for integration with the other study parameters.

### 2.10. Statistical Analysis

All data were analyzed using SPSS Statistics version 21 (SPSS Inc., Chicago, IL, USA). The suitability of continuous variables to the normal distribution was evaluated using visual (histogram and probability graphs) and analytical methods (Kolmogorov–Smirnov and Shapiro–Wilk tests). In the descriptive statistics section, categorical variables are given as numbers and percentages, data that are suitable for the normal distribution of continuous variables are given as arithmetic means and standard deviations, and data that are not suitable for the normal distribution are provided as median (minimum–maximum) values. An independent sample *t*-test was used for comparative analysis between two separate groups of data that matched a normal distribution. The Mann–Whitney U test was used for data that did not match. For comparative analysis of categorical variables between independent groups, the appropriate one from the chi-square (χ^2^) test or Fisher’s exact test was selected. Receiver operating characteristic (ROC) curve analysis was performed to evaluate the performance of TRP and calprotectin in predicting IBD. Multinomial logistic regression identified the independent predictors of disease activation. ROC curve analysis was performed to evaluate the predictive performance of serum TRP, fecal calprotectin, the TRP/CRP ratio, and the KYN/TRP ratio in predicting disease activation in IBD. The analysis assessed the sensitivity, specificity, area under the curve (AUC), positive predictive value (PPV), and negative predictive value (NPV) of these parameters.

## 3. Results

### 3.1. Clinicodemographic Findings

A total of 122 patients were enrolled in the study; however, 6 patients were excluded due to incomplete data. Thus, 116 patients were included in the analysis; 77 (66.4%) had UC, and 39 (33.6%) had CD. Of the patients, 43.1% (*n* = 50) were female and 56.9% (*n* = 66) were male. The median age of the women was 42.07 ± 13.39 years, and the median age of the men was 43.89± 14.64 years. In the examination, according to disease activity, a total of 61 (52.6%) patients were active, including 35 (63.6%) UC and 20 (36.4) CD in total, and 55 (47.4%) in remission: 42 (68.9) UC and 19 (31.1%) CD. Calprotectin, sedimentation, CRP, white blood cell (WBC), and platelet (PLT) levels were significantly higher in patients in remission than in patients in the active period (*p* < 0.001, *p* < 0.001, *p* < 0.001, *p* = 0.001, respectively). Albumin, protein, HB, KYNA, TR, and PA levels were significantly lower in patients in active cycles than in those in remission (*p* < 0.001, *p* = 0.020, *p* < 0.001, *p* = 0.034, *p* < 0.001, and *p* = 0.020, respectively). There were no statistically significant differences between the groups in terms of QA, 3OHKYN, KYN, or ferritin potassium levels (*p* = 0.895, *p* = 0.625, *p* = 0.132, *p* = 0.430, and *p* = 0.189, respectively). Comparative analyses of the biochemical parameters according to disease activity are summarized in Table 1.

Table 2 presents the distribution of patients based on the **Montreal Classification** of **CD** and **UC**.

### 3.2. Comparison of Biochemical Parameters According to Disease Activity

TRP was negatively correlated with ESR, CRP, PLT, WBC, and neutrophil count, while it was positively correlated with total protein and albumin. No correlation was found between TRP and ferritin levels.

A positive correlation was detected between calprotectin and ESR and CRP, PLT, WBC, and neutrophil counts, whereas calprotectin was negatively correlated with albumin and total protein levels. No statistically significant correlation was found between the calprotectin and ferritin levels. Comparative analyses of the biochemical parameters according to disease activity are summarized in Table 3.

### 3.3. The Evaluation of the Relationship Between Tryptophan Metabolites, Calprotectin, and Serum Inflammatory Markers

Diagnostic accuracy analysis showed that the TRP/CRP ratio had the highest predictive value for disease activation, with an AUC of 0.847, demonstrating strong sensitivity (82%) and specificity (73%), making it the most reliable marker. A low TRP/CRP ratio indicates increased inflammation and tryptophan depletion, leading to increased activation of the kynurenine pathway, which contributes to immune dysregulation and is associated with active disease states in both UC and CD. Calprotectin, with an AUC of 0.799, provided high sensitivity (84.2%) but moderate specificity (67.6%), making it useful for detecting disease, but less effective in ruling it out. Serum TRP, with an AUC of 0.711, showed moderate diagnostic accuracy, with a sensitivity of 72.1% and specificity of 62.7%, indicating that it can support diagnosis but should be used alongside other markers. The KYN/TRP ratio had the lowest AUC (0.652), moderate sensitivity (66%), and specificity (53%); however, its high negative predictive value (100%) suggests that it is useful in excluding disease activation when the test is negative. Table 4 presents the results.

### 3.4. Evaluation of Serum TRP and Calprotectin in Terms of Disease Activation Prediction

**Figure 1:**
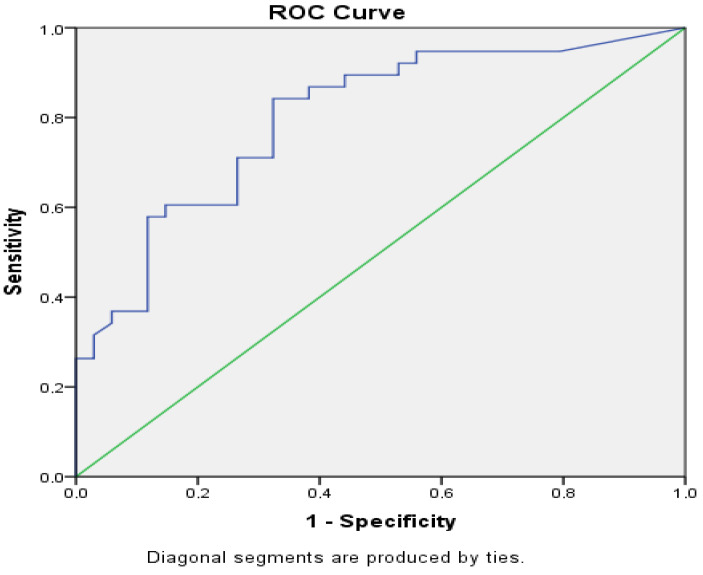
Receiver operating characteristic (ROC) curves for calprotectin (larger results of calprotectin indicate more predictive for disease activation).

**Figure 2:**
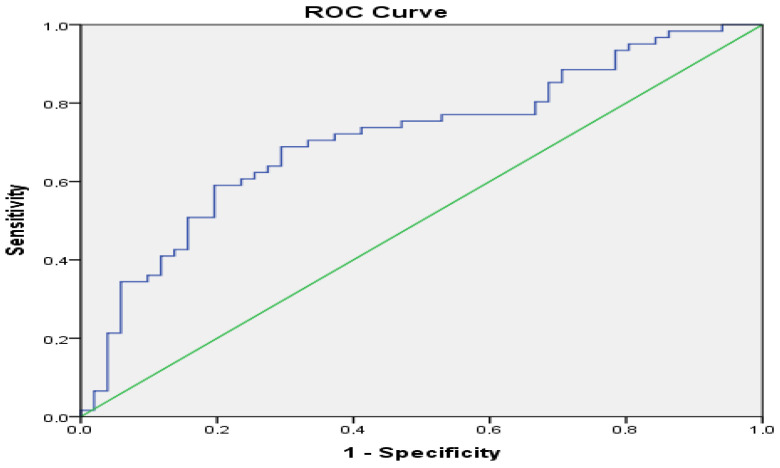
Receiver operating characteristic (ROC) curves for tryptophan (smaller results of tryptophan indicate more predictive for disease activation).

The predictive power of calprotectin and TRP was assessed using ROC analysis. The AUC values and statistical significance of the ROC analysis are presented in Figure 1 and Figure 2.

According to the ROC curve analysis, the sensitivity of TRP to predict disease activation at a ≤11,328.41 ng/mL cut-off was 72.1%, and its specificity was 62.7%. Calprotectin had a cut-off value of ≥89.60 μg/g, a sensitivity of 84.2%, and a specificity of 67.6%.

### 3.5. Multinomial Logistic Regression

#### Independent Predictors

Serum TRP (β = −0.43, *p* < 0.001): Lower levels were associated with increased disease activation. Fecal calprotectin (β = 0.52, *p* < 0.001): Higher levels were strongly associated with disease activation. Both biomarkers were statistically significant predictors of disease activity (*p* < 0.001), indicating strong evidence for their role in IBD monitoring.

### 3.6. Subgroup Analysis

Dichotomized results revealed a higher sensitivity for TRP in patients with UC than in patients with CD. TRP demonstrated a sensitivity of 75% for UC and 65% for CD, suggesting that localized inflammation in UC may drive significant changes in tryptophan metabolism. The AUC for TRP in patients with UC was also higher (0.740) than that in patients with CD (0.670), further highlighting the biomarker’s differential performance. The results are presented in Table 5.

## 4. Discussion

Our investigation revealed that serum TRP levels were diminished, which is consistent with the findings of earlier studies [1,16,17,18]. Notably, our study observed no significant disparity in quinolinic acid levels between the active and remission groups, in contrast to previous studies that reported elevated levels of this TRP breakdown metabolite [19]. This discrepancy highlights the need for further investigation into the metabolic pathways influencing quinolinic acid levels and the underlying mechanisms regulating its production in IBD.

The potential role of tryptophan deficiency in the etiopathogenesis of IBD is noteworthy. Experimental animal models of IBD have demonstrated that tryptophan supplementation can mitigate disease progression [5,6,7,8]. This suggests a need for novel therapeutic approaches based on these findings. In particular, exploring the interplay between tryptophan metabolism, immune modulation, and the gut microbiota offers promising avenues for developing precision-targeted treatments. For example, dietary modifications or probiotic interventions designed to influence tryptophan metabolism may yield novel strategies for IBD management.

Our study explored serum TRP and its metabolites as potential biomarkers for IBD activity, emphasizing their correlation with inflammation and disease progression. Comparatively, Hataysal et al. [20] focused on the kynurenine pathway and oxidative stress markers, revealing reduced TRP and kynurenine levels in patients with IBD. Both studies underscored the role of impaired tryptophan metabolism in IBD pathogenesis. However, while our study primarily analyzed metabolic markers, Hataysal et al. provided a broader perspective by incorporating oxidative stress parameters and offered additional insights into disease mechanisms. These differences highlight the complementary nature of the metabolic and oxidative stress pathways in the pathophysiology of IBD.

Our study also assessed fecal calprotectin and serum TRP levels to evaluate their diagnostic and predictive accuracies for IBD activity. Kunst et al. [20] proposed fecal nervonic acid as a novel biomarker for diagnosing and monitoring IBD, showing significant correlations with fecal calprotectin and CRP. Both studies emphasized the utility of non-invasive biomarkers for disease monitoring. However, our focus on tryptophan metabolism contrasts with the lipid-based biomarker approach of Kunst et al. This distinction highlights the need to investigate diverse metabolic pathways and their diagnostic relevance, potentially enabling a more comprehensive evaluation of disease activity and therapeutic responses.

The importance of microbial and metabolic interactions in IBD was further emphasized in our study through the analysis of tryptophan metabolism. This approach complements the findings of Cisek et al. [21], who investigated gut methanogens and their correlation with disease activity in pediatric patients with IBD. While both studies recognized the significance of gut-derived metabolites, the referenced work focused on microbial composition and its association with fecal calprotectin, whereas our study examined the biochemical markers derived from metabolic pathways. These differences illustrate the value of integrating microbial and metabolic assessments to better understand disease mechanisms and identify novel therapeutic targets.

Finally, our study highlighted the predictive value of fecal calprotectin as a marker of disease activity, demonstrating its diagnostic superiority over serum tryptophan. This finding aligns with the meta-analysis by Shi et al. [22], which confirmed the effectiveness of fecal calprotectin in predicting IBD relapse, identifying an optimal cut-off value of 152 µg/g with high sensitivity and specificity. Although this meta-analysis provides broader statistical validation, our study contributes to the original patient-level data, offering practical insights into the clinical application of fecal calprotectin. Together, these findings reinforce the importance of non-invasive biomarkers for the early detection and monitoring of IBD.

A low TRP/CRP ratio indicates increased inflammation and tryptophan depletion, leading to increased activation of the kynurenine pathway, which contributes to immune dysregulation and is associated with active disease states in both UC and CD. The TRP/CRP ratio offers a balanced approach by combining metabolic and inflammatory markers, providing better insights into disease activity than traditional biomarkers do. The comparison between our study and the AGA Clinical Practice Guideline [14] on biomarkers for UC highlights the potential of the TRP/CRP ratio as a superior diagnostic tool by integrating metabolic and inflammatory markers, offering a more comprehensive assessment of disease activity compared to traditional biomarkers such as fecal calprotectin and CRP. While AGA guidelines recognize fecal calprotectin as a reliable non-invasive marker, they acknowledge its variability due to dietary and systemic influences, whereas our study suggests that TRP/CRP provides better specificity and sensitivity by addressing these limitations. Additionally, both sources emphasize the need for biomarker-based monitoring in clinical practice; however, this study positions TRP/CRP as a promising tool for personalized medicine, potentially reducing the need for invasive procedures. However, further validation in larger cohorts is required to establish standardized cut-off values and confirm its broader applicability in IBD management.

### 4.1. Explaining Low AUC Values for Tryptophan

The observed AUC value of 0.711 for serum TRP underscores its limitations as a standalone biomarker for predicting disease activation in IBD. The variability in TRP levels can be attributed to several external factors, including dietary habits, medication use, and gut microbiota composition, which may influence its metabolic pathways and reduce diagnostic accuracy. Furthermore, TRP is involved in multiple physiological processes beyond inflammation, complicating its interpretation as an inflammatory marker. The absence of a healthy control group in this study further limited the contextual assessment of the diagnostic performance of TRP. However, the integration of TRP with inflammatory markers, such as CRP, in the TRP/CRP ratio has demonstrated improved diagnostic accuracy, suggesting that combining metabolic and inflammatory markers enhances sensitivity and specificity. Future studies, incorporating larger cohorts with healthy controls and evaluating TRP in composite biomarker panels, may further optimize its clinical utility for IBD management.

### 4.2. Applicability to Precision Medicine

A more personalized approach to IBD management can be achieved by incorporating metabolic and inflammatory biomarkers such as the TRP/CRP ratio. The superior diagnostic performance of TRP/CRP allows for the more precise stratification of patients into active or remission states, facilitating targeted therapeutic strategies. This biomarker combination enhances the ability to monitor disease progression and treatment response, aligning with the broader goals of precision medicine by integrating metabolic and inflammatory data for individualized patient care.

### 4.3. Mechanistic Insights of the Kynurenine Pathway

The Kynurenine pathway plays a crucial role in immune regulation by modulating inflammation and maintaining intestinal homeostasis. TRP metabolism through this pathway produces KYN, which interacts with the aryl hydrocarbon receptor (AhR) to promote the production of anti-inflammatory cytokines such as IL-22. Additionally, kynurenine metabolites counteract pro-inflammatory processes by influencing T-cell differentiation and reducing Th17-mediated inflammation. The dysregulation of this pathway, as observed in active IBD, may lead to an imbalance between the pro- and anti-inflammatory responses, exacerbating mucosal damage and disease progression. This mechanistic understanding underscores the importance of targeting the kynurenine pathway for IBD therapy [23].

## 5. Limitations of the Study

The primary limitations of this study include:

- The relatively small sample size may have limited the generalizability and statistical power of our findings. This limitation was largely due to the COVID-19 pandemic, during which stringent restrictions, reduced hospital visits, and the prioritization of resources for COVID-19 care significantly affected patient recruitment and clinical study.
- The absence of a healthy control group restricts the ability to draw comparisons between diseased and non-diseased populations.
- The lack of dietary and medication adjustments may influence TRP levels and create potential confounding factors.
- The limitations of the TRP/CRP ratio as a biomarker for IBD include the lack of standardized cut-off values, which may affect its clinical applicability, and the influence of factors such as diet, gut microbiota composition, and medications on tryptophan metabolism. Additionally, the available studies are limited in sample size and diversity, necessitating further large-scale multicenter trials to establish their reliability and predictive value.

## 6. Clinical Implications

A distinctive feature of our research was its dual focus: elucidating the role of TRP and its metabolites in disease activation, while also seeking a more economical marker for early-stage disease activation than the well-established fecal calprotectin method. Despite our efforts, fecal calprotectin appears to maintain its superiority even in the early stages of the disease. Nevertheless, the potential of TRP and its metabolites as early disease activation markers warrants further exploration in larger patient cohorts, necessitating the development of appropriate methodologies. Advanced analytical techniques, including metabolomic profiling and machine learning algorithms, can refine the predictive value of these biomarkers. Potential therapeutic strategies for managing IBD based on the TRP/CRP ratio focus on nutritional and pharmacological intervention. Nutritional approaches include tryptophan-rich diets or supplementation to restore TRP levels and improve gut homeostasis, whereas probiotics that enhance TRP metabolism through the indole pathway may offer additional benefits. Pharmacological strategies target the kynurenine pathway using inhibitors, such as IDO1 inhibitors, to prevent excessive TRP depletion and immune dysregulation. These interventions aim to restore the TRP metabolism balance, reduce inflammation, and improve disease outcomes in patients with IBD.

## 7. Conclusions

The TRP/CRP ratio has emerged as a superior biomarker for predicting disease activation in IBD, offering a more comprehensive assessment by integrating the metabolic and inflammatory pathways. Although fecal calprotectin remains a well-established marker, the TRP/CRP ratio provides enhanced diagnostic accuracy and better reflects systemic inflammation and metabolic disturbances. Its ability to identify active disease states with high sensitivity and specificity makes it a valuable tool for clinical decision making. Future research should focus on validating the TRP/CRP ratio in larger cohorts and exploring its potential role in personalized treatment strategies for IBD.

## Figures and Tables

**Table 1:** Comparison of clinical and laboratory parameters according to disease activation.

	Active *n* = 61	Remission *n* = 55	*p*
Age, year	40.50 ± 12.90	45.45 ± 14.57	0.055
Gender			
Female	29 (47.5)	21 (38.2)	0.309
Male	32 (52.5)	34 (61.8)	
Disease type			
Ulcerative colitis	42 (68.9)	35 (63.6)	0.164
Crohn disease	19(31.1)	20 (36.4)	
ESR, h	36 (3.82–125.0)	18 (3–137.0)	**<0.001**
CRP	12.35 (1.24–241.0)	5.00 (1.00–74.50)	**<0.001**
WBC	8460 (3400–22,620)	6955 (4550–12,590)	**<0.001**
HB, g/dL	12.40 (5.90–17.40)	14.10 (8.00–18.10)	**<0.001**
PLT, 10^3^	329 (142–608)	260 (165–747)	**0.001**
Neutrophile	5480 (1770–18450)	4105 (2540–7760)	0.187
T. protein	7.30 (3.50–8.40)	7.50 (4.30–8.40)	**0.020**
Albumin	3.95 (1.40–5.00)	4.40 (1.90–5.00)	**<0.001**
Ferritin	20.00 (1–715.00)	26.00 (5.00–325.00)	0.430
Calprotectin, µg/g	2.10 (0.80–4.80)	1.90 (1.20–3.30)	**<0.001**
PA, ng/mL	5.73 (0.35–80.77)	14.13 (0.35–133.37)	**0.022**
KYNA, ng/mL	32.17 (3.02–205.04)	54.96 (5.20–162.87)	**0.034**
3OH KYN, ng/mL	32.87 (7.37–173.84)	30.80 (13.25–132.33)	0.625
KYN ng/mL	464.33 (47.21–1048.05)	545.99 (129.24–1155.75)	0.132
TRP, ng/mL	9667.06 (1210.71–15,703.02)	11,916.08 (16.42–16,104.68)	**<0.001**
QA, ng/mL	49.68 (3.71–332.33)	47.96 (9.67–281.67)	0.895
**Treatment**			
5-ASA or budenosid	22 (33.8)	32 (64.0)	**0.027**
Steroid	9 (13.8)	2 (4.0)	**0.034**
Azatiopurine	18 (27.7)	8 (16.0)	0.112
Anti-TNF	15 (23.1)	8(16.0)	0.215
Vedolizumab	1 (1.5)	0 (0.0)	0.315

Abbreviations: ESR: erythrocyte sedimentation rate; CRP: C-reactive protein; WBC: white blood cell count; HB: hemoglobin. PLT: platelet; T. protein: total protein; PA: Pyridoxic Acid; KYNA: Kynurenic Acid; 3OH KYN: 3-Hydroxykynurenine; KYN: kynurenine; TRP: tryptophan; QA: Quinolinic Acid; 5-ASA: 5-Aminosalicylic Acid; Anti-TNF: anti-tumor necrosis factor.

**Table 2:** Patient Distribution According to Montreal Classification.

	Montreal Classification	Number of Patients	Total
Crohn’s Disease	L1	15	39
	L2	3	
	L3	21	
Ulserative Colitis	E1	14	77
	E2	23	
	E3	40	

**Table 3:** An evaluation of the relationship between serum inflammatory markers and serum TRP and fecal calprotectin.

	Tryptophan		Calprotectin	
	r	*p*	r	*p*
ESR	**−0.540**	**<0.001**	**0.455**	**<0.001**
CRP	**−0.434**	**<0.001**	**0.431**	**<0.001**
WBC	**−0.254**	**0.008**	**0.263**	**0.029**
PLT	**−0.520**	**<0.001**	**0.452**	**<0.001**
Neutrophile	**−0.271**	**0.005**	**0.306**	**0.011**
Ferritin	−0.144	0.175	0.075	0.574
T. protein	**0.408**	**<0.001**	**−0.264**	**0.032**
Albumin	**0.727**	**<0.001**	**−0.487**	**<0.001**

Abbreviations: ESR: erythrocyte sedimentation rate; CRP: C-reactive protein; WBC: white blood cell count; PLT: platelet; T. protein: total protein.

**Table 4:** Performance of serum tryptophan and calprotectin for prediction disease activation.

Variable	AUC	*p*	Cut-Off	Sensitivity	Specificity	PPV	NPV
	(95% CI)						
Tryptophan	0.711 (0.615–0.8079)	**<0.001**	≤11,328.41	72.10%	62.70%	79.40%	71.20%
Calprotectin	0.799 (0.697–0.902)	**<0.001**	≥89.60	84.20%	67.60%	86.00%	61.10%
TRP/CRP	0.847(0.772–0.992)	**<0.001**	≤1630.2935	82%	73%	81.80%	77/6 %
KYN/TRP	0.652(0.549–0.754)	**0.007**	≥0.0459	66%	53%	58.60%	100%

Abbreviations: CRP: C-reactive protein; KYN: kynurenine; TRP: tryptophan.

**Table 5:** Subgroup Analysis Performance of Tryptophan.

	Ulcerative Colitis (UC)	Crohn’s Disease (CD)
Sensitivity (%)	75	65
AUC	0.740	0.670

## Data Availability

The data presented in this study are available upon request from the corresponding author.

## Supplementary Materials

The following supporting information can be downloaded at: https://www.mdpi.com/article/10.3390/jcm14031016/s1, Supplementary S1. Chromatographic and mass spectrometric conditions for the analysis of tryptophan metabolites. The table includes system specifications, mobile phase composition, flow rate, injection volume, and detection parameters using TSQ Quantum Access MAX Triple Stage Quadrupole Mass Spectrometry. Validation parameters such as linearity, recovery, limit of detection (LOD), limit of quantification (LOQ), repeatability, and intermediate imprecision are also provided.
