# Supplementary material for: Evaluation of Tryptophan and Its Metabolites in Predicting Disease Activation in Inflammatory Bowel Disease
Source: J Clin Med. 2025 Feb 5;14(3):1016. doi: 10.3390/jcm14031016 (PMC11818851; doi:10.3390/jcm14031016)
Supplement: Supplementary file 1 [file jcm-14-01016-s001.zip › jcm-3408255-supplementary.pdf]

Supplement S1:

Chromatographic Condition

System:	Dionex UltiMate™ 3000 UHPLC
Column and Dimensions:	Thermo Hypersil Gold 2.1 × 10mm, 3 µm
Mobile Phases:	A) Su (0.1 % formic acid)<br>B) Methanol Su (0.1 % formic acid)
Flow Rate:	0.3 mL/min
Temperature:	15 °C
Injection:	10 µL,

Mass Spectrometric Condition

System:	TSQ Quantum Access MAX Triple Stage Quadrupole Mass
Ion Source:	Electrospray Ionisation (ESI)
Spray Voltage:	3000 V
Vaporizer Temp.:	450 ºC
Capillary Temp.:	180 ºC
Sheath Gas:	45 arbitrary units
Auxiliary Gas:	30 arbitrary units
Detection Mode:	SRM
Collition gas pressure (mTorr):	1.5
TChrom FİIter Peak Width (s)	40

Mobile-phase gradient contitions

Time (min)	Flow (ml/min)	%A	%B
0	0.300	90	10
0	0.300	90	10
4	0.300	90	10
5	0.300	40	60
13	0.300	15	85
13	0.300	90	10
18	0.300	90	10

SRM and parameters

	Procursor (m/z)	Product (m/z)	Collition energy(V)	Tube Lens
Tryptophan	205.1	118.1	40	100
Tryptophan-d5	210.1	121.3	40	100
Kynurenine	209.1	94.1	18	50
Kynurenine -d4	213.1	98.1	18	50
3 Hydroxykynurenine	281.1	110.1	18	50
Kynurenic acid	190.0	116.0	30	50
Kynurenic acid-d5	195.1	121.1	30	50
Picolinic acid	180.1	96.04	22	50
Picolinic acid-d3	184.1	100.1	22	50
Quinolinic acid	280.0	124.0	20	50
Quinolinic acid-d3	283.0	127.1	20	50

Validation Parameters (22,23,24):

**Performance Charecteristic**	Procedure	**TRP**	**KYN**	**3OHKYN**	**KYNA**	**PA**	**QA**
**Linearity**	Ref 23.	30000	2500	1000	1000	1000	1000
**Recovery (%)**	3 spiked samples, 2 levels	92	94	102	91	104	106
**LOD (ng/ml)**	S/N 3SD	18.9	12.1	2.1	1.5	0.6	1.1
**LOQ (ng/ml)**	S/N 10SD	48.4	27.3	4.8	3.9	1.8	2.4
**Repeatebility**	3 samples repeated , 10 times in one day	<6.4	<8.7	<11.3	<11.6	<7.2	<10.8
**Intermediate Imprecision**	3 samples repeated, 10 times in 10 day	<9.2	<12.5	<13.7	<13.5	<12.9	<14.4
